# Gene set-based module discovery in the breast cancer transcriptome

**DOI:** 10.1186/1471-2105-10-71

**Published:** 2009-02-26

**Authors:** Atsushi Niida, Andrew D Smith, Seiya Imoto, Hiroyuki Aburatani, Michael Q Zhang, Tetsu Akiyama

**Affiliations:** 1 Laboratory of Molecular and Genetic Information, Institute of Molecular and Cellular Biosciences, The University of Tokyo, 1-1-1, Yayoi, Bunkyo-ku, Tokyo, 110-0032, Japan; 2 Cold Spring Harbor Laboratory, Cold Spring Harbor, NY 11274, USA; 3 The Institute of Medical Science, The University of Tokyo, 4-6-1 Shirokanedai, Minato-ku, Tokyo 108-8639, Japan; 4 Genome Science Division, Research Center for Advanced Science and Technology, The University of Tokyo, 4-6-1 Komaba, Meguro, Tokyo, 153-8904, Japan

## Abstract

**Background:**

Although microarray-based studies have revealed global view of gene expression in cancer cells, we still have little knowledge about regulatory mechanisms underlying the transcriptome. Several computational methods applied to yeast data have recently succeeded in identifying expression modules, which is defined as co-expressed gene sets under common regulatory mechanisms. However, such module discovery methods are not applied cancer transcriptome data.

**Results:**

In order to decode oncogenic regulatory programs in cancer cells, we developed a novel module discovery method termed EEM by extending a previously reported module discovery method, and applied it to breast cancer expression data. Starting from seed gene sets prepared based on *cis*-regulatory elements, ChIP-chip data, and gene locus information, EEM identified 10 principal expression modules in breast cancer based on their expression coherence. Moreover, EEM depicted their activity profiles, which predict regulatory programs in each subtypes of breast tumors. For example, our analysis revealed that the expression module regulated by the Polycomb repressive complex 2 (PRC2) is downregulated in triple negative breast cancers, suggesting similarity of transcriptional programs between stem cells and aggressive breast cancer cells. We also found that the activity of the PRC2 expression module is negatively correlated to the expression of EZH2, a component of PRC2 which belongs to the E2F expression module. E2F-driven EZH2 overexpression may be responsible for the repression of the PRC2 expression modules in triple negative tumors. Furthermore, our network analysis predicts regulatory circuits in breast cancer cells.

**Conclusion:**

These results demonstrate that the gene set-based module discovery approach is a powerful tool to decode regulatory programs in cancer cells.

## Background

In the last decade, microarray technology has produced exploding amounts of cancer transcriptome data; especially, breast cancer transcriptome has been intensively profiled. Human breast tumors show diversity in their histology, prognosis, and responsiveness to treatments. The microarray technology has demonstrated that transcriptomic diversity underlies phenotypic diversity, and brought great progress in our molecular understanding of breast cancer [1]. However, compared with the increasing knowledge about the transcriptome, little is yet known about regulatory programs generating the transcriptomic diversity.

To decode gene regulatory programs controlling the breast cancer transcriptome, we searched for *cis*-regulatory motifs associated with tumor phenotypes in our previous study [2]. One of the limitations of this method is that it takes a supervised approach and requires sample information. In this study, we introduce an alternative method which focuses on expression modules and does not require sample information. An expression module is defined as a set of coexpressed genes controlled by a common regulatory mechanism. Although expression modules were originally visualized by clustering analysis of microarray data [3], methods based only on expression data are insufficient to reveal regulatory programs controlling such expression modules. Recently, approaches that combine expression data and *cis*-regulatory information have succeeded in identify gene regulatory programs of lower organisms like *Saccharomyces cerevisiae *[4,5]. However, such module discovery approaches have rarely been applied to cancer transcriptome data, although a number of analyses based on prescribed sets of genes have also been performed in order to analyze oncogenic regulatory programs [6,7].

Our new computational method termed EEM (Extraction of Expression Modules) is constructed for extracting expression modules in the cancer transcriptome. Our approach is based on an integrative method by Bar-Joseph et al. [5], which successfully identified yeast expression modules by integrating ChIP-chip and expression data. By combining with gene set-based approaches [6,7], we extended their approach and made it applicable to cancer transcriptome data. Starting from seed gene sets predicted based on *cis*-regulatory elements, ChIP-chip data, and gene locus information, EEM statistically evaluates their functionality and refines them based on their expression coherence. We analyzed breast cancer microarray data by EEM, and find 10 expression modules in the breast cancer transcriptome. Our additional bioinformatics analysis validated the 10 expression modules and demonstrated their significance in the pathophysiology of breast cancer.

## Methods

### Methods Overview

The EEM algorithm discovers an expression module by combining two types of data: seed gene sets and expression profile data. A set of genes whose expressions are considered to be regulated by the same molecular mechanism could be predicted based on various types of data, and prepared as a seed gene set. EEM assesses functionality of the seed gene set based on expression coherence. If seed gene set functions as expression module, all genes in it are ideally expressed coherently. Although a functional seed gene set might include false positives, or non-functional module genes in the biological context of interest, at least a significant fraction of seed genes should behave coherently in the expression data. This assumption is verified by the observation that putatively functional gene sets often harbors a large cluster of genes which behave coherently (see Additional File 1). EEM extracts only such a coherently expressed gene subset, filtering out false positive or non-functional module genes. Taking a geometric approach, EEM searches for the largest subset with a minimum degree of coexpression (specified by radius parameter *r*). Concurrently, EEM statistically evaluates the size of the retrieved coherent subset using a Z score based on randomization tests. If the Z score is greater than the prespecified cutoff value, we conclude that the seed gene set includes a functional expression module and the coherent subset is extracted as an expression module. We observe that the expression modules extracted by EEM are more functionally enriched than seed gene sets. This observation would justify our refinement procedure (see Additional File 1).

**Figure 1:**
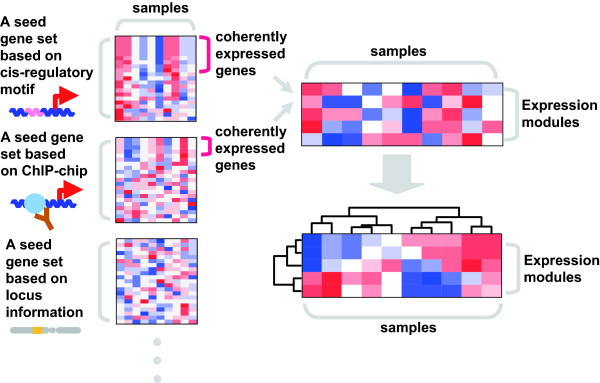
**Schema of systematic search for expression modules**. We prepared a collection of seed gene sets based on *cis*-regulatory motifs, ChIP-chip and gene locus information. We next statistically evaluated whether each seed gene set includes a significant large number of coherently expressed genes in expression profile data. If such a coherently expressed gene subset exists, we assumed it as an expression module, and obtained its averaged expression profile as an activity profile.

Employing this EEM algorithm, we systematically searched for expression modules in the breast cancer transcriptome (Figure 1). In our search, a collection of seed gene sets are prepared based on *cis*-regulatory motifs, ChIP-chip data, and gene locus information. Since genes that possess a common *cis*-regulatory element in their promoters could be regulated by a common transcription factor (TF), we can predict an expression module based on the *cis*-regulatory motif. We searched human gene promoters and its mouse homolog promoters for 200 motif using PWMs obtained from the TRANSFAC and JASPAR databases [8,9], and prepared 200 seed gene sets which include genes with common motifs in their promoters. We can also predict expression modules utilizing ChIP-chip data, which provide direct evidence of TF binding in the *cis*-regulatory regions. Published ChIP-chip results [10-14] are collected to prepare seed gene sets. DNA copy number alteration is known to have a significant effect on the cancer transcriptome as well as transcriptional regulation [15]. Hence, we also regarded it as one of expression regulatory mechanisms in cancer cells. Genes residing in a chromosomal region which is subjected to copy number alteration could be expressed coherently, and viewed as an expression module; taking sliding window approach, we prepared seed gene sets which consist of genes residing on the same chromosomal region. For each of the prepared seed gene sets, we tested the presence of coherently expressed gene subsets in breast cancer microarray data [16]. If such coherently expressed genes exist, we then extracted them as an expression module. Furthermore, the average expression profile of the predicted expression module can be considered as its activity in each of tumor samples. The expression module activity profiles were then analyzed using ordinary methods applied to gene expression profile data like clustering and survival analysis.

This approach is an extension of a module discovery method, GRAM, which is developed by Bar-Joseph et al. [5] for learning yeast expression modules from microarray and ChIP-chip data. In the first step, GRAM uses ChIP-chip data to find a small number of genes whose upstream regions are bound by common TFs with high confidence. In the second step, the microarray data is used to extract a coherently expressed subset of these genes. Finally, the resulting set is expanded by adding genes which are identified to be bound by the TFs with less strict criteria. Although GRAM and another similar method [17] require binding P values in ChIP-chip data, we relaxed this requirement by taking a gene set-based approach. Although gene set-based approaches are simpler than direct integration of ChIP-chip and expression data, they have shown substantial successes in cancer transcriptome analysis [6,7]. From ChIP-chip data, we prepared gene sets as seed gene sets, by retrieving genes which have binding sites within specified *cis*-regulatory regions and with P values below a specified threshold. In addition to ChIP-chip data, our analysis utilized *cis*-regulatory motifs and locus information to generate gene sets, because available human ChIP-chip data are insufficient for comprehensive analysis and they also provide clear evidence of transcriptional regulation or genomic alteration. Although EEM also takes a module discovery approach similar to GRAM, there are some clearly different points. In contrast to GRAM, EEM starts from a sufficient number of genes that are predicted to be under common regulatory mechanism, and refines them to produce a final expression module utilizing expression profiles. In this process, EEM evaluates statistical significance of the identified expression module by measuring how many of module genes are coherently expressed in the expression data. This statistical evaluation based on the expression coherence is a novel feature which is not implemented by other module discovery methods.

EEM is also regard as one of gene set screening methods like Gene Set Enrichment Analysis (GSEA) [18]. GSEA screens for gene gets that have a significant bias in a ranked list according to their differential expressions between two sample groups, while our approach searches for significant gene sets based on their expression coherence. However, because GSEA takes a supervised approach which uses sample labels, it potentially fails to identify expression modules which do not correlate with sample labels. By contrast, EEM realizes an unsupervised analysis, which does not depend on sample information and can search for expression modules more globally.

As we mentioned above, our method finally produces activity profiles of expression modules. Because microarray data usually include expression profiles of thousands of genes, it is difficult to understand the raw data intuitively. On the other hand, since our activity profiles consist of those of a small number of expression modules, they provide concise description of the transcriptome that allows it to be understood more easily. This problem can also be solved using dimension reduction approach of gene expression data. Dimension reduction is originally addressed by a study utilizing singular value decomposition [19], and can be performed by many other methods [20-23]. However, because most of them are based on purely mathematical framework, deduced components do not necessarily have biological meanings and are often difficult to understand biologically. By contrast, since expression modules deduced by EEM are derived from biologically meaningful seed gene sets, they can always be associated with molecular mechanisms. By extracting module modules as biologically meaningful components in expression data, EEM provides intuitively understandable views of transcriptomes.

### EEM algorithm

Let *E *= {*e*_1_,...,*e*_*n*_} be a set of gene expression profiles such that each *e*_*i *_∈ *E *is a vector *e*_*i *_= (*e*_*i*1_,...,*e*_*im*_) of values with *e*_*ij *_giving the expression of the *i*-th gene in the *j*-th condition (or sample). Each *e*_*i *_∈ *E *then exists as a point in a continuous *m*-dimensional gene expression space S. Although the expression values can be obtained by any means, we may assume they are from gene expression microarray experiments. EEM operates on a subset *E*_*M *_⊆ *E *called the seed gene set (we describe below how such seed gene sets are obtained). For a given radius *r *and point *x *∈ S, define

(1) *C*_*x *_= {*e*_*i *_∈ *E*_*M *_: *d*(*e*_*i*_, *x*) ≤ *r*},

where *d *is the Euclidean distance. We call *C*_*x *_the coherently expressed gene set (or simply *coherent set*), and the point *x *is called the *center *of *C*_*x*_. The objective of EEM is to find maximal sized coherent set *C*_*B *_(and corresponding center *B*) for the genes in *E*_*M*_. We remark that the center *B *may not necessarily correspond to any profiles in *E*_*M*_. We also call *B *the *activity profile *for genes in *C*_*B*_. As stated above, the distance measure we use to define a degree of co-expression between genes is Euclidean distance. In practice the expression profiles are normalized, so this is equivalent to measuring similarity using Pearson correlation. Our method is intended for large datasets (based on microarray expression profiles), and employs a heuristic modified from a previously proposed algorithm [5]. Similar geometric optimization problems arise in the context of clustering [24,25]. EEM attempts to find an optimal center for *E*_*M *_in two stages. The first stage identifies a candidate center *B*_1 _from among the expression profiles in *E*_*M*_. For each *e*_*i *_∈ *E*_*M*_, the set $C_{e_{i}}$ is constructed (see Equation 1). The profile *e*_*i *_∈ *E*_*M *_with maximal |$C_{e_{i}}$| is retained as *B*_1_. The second stage uses *B*_1 _to find an improved center. Let *T *⊆ *E*_*M *_denote the set containing the 9 profiles in *E*_*M *_closest to *B*_1 _along with *B*_1 _itself (*i.e*. |*T*| = 10). For each triple {*t*_1_, *t*_2_, *t*_3_} ⊂ *T*, the mean profile *t *= (*t*_1 _+ *t*_2 _+ *t*_3_)/3 is constructed and *C*_*t *_is evaluated. The mean profile *t *that maximizes |*C*_*t*_| over all triples from *T *is retained and returned by EEM as the optimal center *B *along with the identity of genes in *C*_*B *_(see the Appendix section for a pseudocode for this optimization procedure).

EEM includes a critical step to estimate the statistical significance of the size of the coherent set, given the full set of expression profiles from the expression data set (recall that the procedure described above operates on a subset *E*_*M *_⊆ *E *defined by a seed gene set). This is accomplished by sampling subsets of size *k *uniformly at random from the full set *E *of expression profiles, where *k *= |*E*_*M*_|. The EEM optimization procedure (described above and summarized as in the Appendix section) is applied to each sampled subset to produce an empirical distribution for the sizes of coherent sets derived from *E*. The mean and standard deviation from this empirical distribution are used to obtain a *Z *score for |*C*_*B*_| corresponding to *E*_*M*_, and *Z *score threshold is used to determine whether a particular coherent set is significant. Our results are based on *Z *scores estimated using 500 randomly sampled subsets of expression profiles.

### Preparation of Expression data

From GEO database, we downloaded Affymetrix GeneChip data of 252 breast tumor samples [16] (the accession number is GSE3494). Absolute expression values of a data set were converted to the logarithmic scale and normalized so that the mean is equal to 0 and the variance is equal to 1 in each sample. The Probe set IDs were converted to Ensembl gene IDs. In cases that one gene ID matches multiple probe set IDs, the probe set which shows the most variance among the samples was mapped to the gene. A variation filter was then applied to the data, and we obtained 5000 genes with the highest variance. The expression profiles of the 5000 genes were normalized across samples and subjected to the following analysis.

### Preparation of seed gene sets

#### Preparation based on *cis*-regulatory motifs

We prepared promoter data of human genes and mouse genes from the Ensembl database (Release 44). Assuming TSSs (transcription start sites) as gene starts registered in Ensembl, a repeat-masked promoter sequence covering the 500 bp upstream and 100 bp downstream of the TSS for each gene was retrieved from the genome sequences.

As *cis*-regulatory motif data, we prepared PWMs (position weight matrices). The value *f*_*ib *_of a PWM represents the frequency of nucleotide base *b *at the *i*-th position in a motif. The frequencies of bases in each position are normalized so that ∑_*b*∈{*a*, *t*, *g*, *c*}_*f*_*ib *_= 1. If *f*_*ib *_= 0, we reassigned *f*_*ib *_= 0.001 to avoid errors in log calculations. We acquired a total of 601 PWMs, which consist of vertebrate 513 PWMs annotated as "good" in TRANSFAC 10.1 [8] and 88 PWMs from JASPAR core [9]. We then removed extremely simple or complex PWMs based on their information contents to produce a set of total 511 PWMs whose information contents range from 5 to 15. The information content *R *of a PWM is defined as follows:

$$R=2w-\sum_{i=1}^{w}H_{i},$$

where *w *is the width of the motif, and *H*_*i *_is the information entropy at the *i*-th position defined by

$$H_{i}=-\sum_{b=a,c,g,t}f_{ib}\log_{2}f_{ib}.$$

Since this set includes highly redundant PWMs, they were subjected to clustering to reduce the redundancy. For clustering, the dissimilarity between two PWMs *A *and *B *was calculated based on the Kullback-Leibler divergence. At every alignment offset, the PWMs were extended using a column representing the uniform base frequency (*f*_*ib *_= 0.25 for all *b*) so that all position of two aligned motifs were matched. As for this alignment step, we followed a method used by Xie et al. [26] For every pair of the extended PWMs, *A' *and *B'*, whose length are *w'*, the dissimilarity *D*_*A*'*B*' _is calculated by:

$$D_{A^{\prime }B^{\prime }}=\sum_{i=1}^{w^{\prime }}\sum_{b=a,t,g,c}(f_{ib}^{A^{\prime }}-f_{ib}^{B^{\prime }})\log\frac{f_{ib}^{A^{\prime }}}{f_{ib}^{B^{\prime }}}.$$

We assumed the lowest score of *D*_*A*'*B*' _as the dissimilarity between *A *and *B*, *D*_*AB*_. Note that *D*_*AB *_= *D*_*BA *_holds. Using the partition around medoids algorithm, the 511 PWMs are divided into 200 clusters. We used 200 medoids of the clusters in the following analyses.

To predict expression modules, we searched promoter sequences for TF binding motifs based on the log odds ratio *L *between a PWM and background base frequency $f_{b}^{bg}$. Using the STORM program [27], we calculated log odds ratio *L*_*s *_for every subsequence of each promoter *s *(including the complementary strand), whose length is equal to the width of the motif of interest, *w*:

$$L_{s}=\sum_{i=1}^{w}\log\frac{f_{ib_{i}}}{f_{b_{i}}^{bg}}.$$

In our analyses, $f_{b}^{bg}$ is the base composition of each promoter, and the maximum of *L*_*s *_in a human promoter sequence was taken as the motif score *L*^human ^for the sequence. For human genes whose mouse homolog is registered in Ensembl, *L*^mouse ^was also calculated. *L*^human ^and *L*^mouse ^were then averaged to produce the final motif score *L*. For human genes that do not have any homologs, we used *L*^human ^as *L*. Among all genes analyzed, genes which score the 5% highest *L *were assumed as a seed gene set regulated by the motif. For each of the 200 PWMs, we performed this procedure to produce 200 seed gene sets.

#### Preparation based on ChIP-chip data

We obtained TF-bound gene sets identified in ChIP-chip experiments; CREB1, FOXA2, HINF1, HNF4, HNF6 and USF1 in hepatocyte [13], and NF*κ*B in U931 cells [11]. We assumed genes which are bound by EED and SUZ12, and trimetylated at histone H3 lysin-27 in ES cells [12] as a PRC2-bound gene set. A p53-bound gene set was obtained from [14]. For ER-bound genes, we analyzed ChIP-chip results [10], and retrieved genes which are bound by ER in their promoters (from the TSSs to 3 kbp upstream), 5'UTRs or first introns with P values of < 10^-50^.

#### Preparation based on locus information

A sliding window was employed to prepare seed gene sets consisting of genes that reside on the same chromosomal region. A 10 Mb window was slid along each chromosome at interval of 1 Mb. At each position, if the window includes more than or equal to 10 genes, we pooled them in the gene set library and obtained 2766 seed gene sets.

### Setting of parameters

#### The radius parameter

To specify the radius parameter *r *in EEM, we converted the absolute distance to a relative distance for each expression data set. Expression spaces specified by different data sets have different dimensions and different densities of points. Therefore, instead of the absolute distance, we used a relative distance which practically acts as an equal measure for different data sets. To convert the absolute distance, *d*^absolute ^to such a relative distance, *d*^relative^, we define coherent set $C_{x}^{\text{all}}$ for all *e*_*i *_∈ *E*, similarly to Equation 1:

$$C_{x}^{\text{all}}=\{e_{i}\in E:d(e_{i},x)\leq r\},$$

where *r *is a given radius parameter and point *x *∈ S. The maximal sized coherent set $C_{B}^{\text{all}}$ can also be found based on the above-described algorithm for radius *r *= *d*^absolute^. *d*^relative ^is then defined as follows:

$$d^{\text{relative}}=\frac{\left|C_{B}^{\text{all}}\right|}{\left|E\right|}.$$

In our analysis, we assumed *r *= 0.05 in the relative distance. It should be should noted that we also tried to use *r *= 0.03 and 0.10 and observed that the identified expression modules are essentially the same; although the number of module genes increases as *r *increases, statistical significance, activity profiles and enriched GO terms were essentially unchanged. Exceptionally, we used a larger radius for the PRC2 expression module (see Additional File 1).

#### The threshold of Z scores

We assumed that a seed gene set includes a functional expression module, if its *Z *score is greater than a threshold. In this paper, we set 4.0 as the threshold. The reason why we set 4.0 as the threshold is that when we permuted gene labels in microarray data, no expression modules showed greater *Z *scores than 4.0. Therefore, we concluded that this threshold is sufficiently conservative and the resulting expression modules are expected to have high accuracy.

### Evaluation of obtained expression modules

#### Gene ontology analysis

We evaluated the enrichment of GO categories in each identified expression module by using GO::TermFinder [28]. The GOA file we used was obtained from EBI. To predict biological function for each expression module, we also reported the GO category scoring the lowest P values as the most enriched GO.

#### Survival analysis

Kaplan-Meier survival curves were obtained for two patient groups with high or low activity of each identified expression module. The cutoff of the high and low groups was optimized to achieve the most significant P value in the Kaplan-Meier analysis with at least 20% patients at each group. Since the optimized P values in the Kaplan-Meier analysis overestimate the significance, we reported P values based on Cox regression analysis.

#### Network analysis

We evaluated enrichment of physically interacting gene pairs in the expression modules based on PPI data obtained from the Human Protein Reference Database (HPRD) [29]. To calculate a Z score for the number of interacting pairs in an expression module, we randomly sampled 500 gene sets with the same number of genes of the considered expression module. After finding expression modules having the significant number of PPIs, we constructed PPI subnetworks for elucidating molecular circuits. The PPI subnetworks were constructed by the interacting protein pairs and their first neighbor in PPI data. The network visualization was performed using CytoScape [30].

## Results and discussion

Our systematic search identified 10 expression modules in the breast cancer transcriptome (Table 1). Based on *cis*-regulatory motifs, we identified expression modules regulated by E2F, NFY, RUNX, IRF, and ETS family TFs. Hereafter, we use the TF name to refer to the family, e.g. the E2F module will refer to the module associated with the E2F family of TFs. ChIP-chip data led us to identify expression modules regulated by the estrogen receptor (ER), Polycomb repressive complex 2 (PRC2), and NF*κ*B. In addition to these transcriptional modules, incorporation of locus information yielded two expression modules, which are located on the 17q12 and 8q24 locus. The reproducibility of these results was confirmed by analysis using other independent microarray data [31] (see Additional File 1).

**Table 1:** Motif associated with histological grades or prognosis identified based on independent datasets

Module ID	Size	Z score	The most enriched GO	P value for the most enriched GO	Z score for nearest neighbor pair	Z score for nearest or next to nearest pairs
ETS	47	10.0	immune response	4.55 × 10^-15^	10.9	9.85
IRF	47	7.59	immune response	6.19 × 10^-12^	N.S.	3.69
E2F	37	6.67	cell cycle	1.98 × 10^-20^	32.5	24.5
RUNX	34	5.57	immune response	2.57 × 10^-11^	12.1	6.08
NFY	30	4.22	cell cycle	9.48 × 10^-14^	15.4	12.3
NF*κ*B	29	9.53	immune response	1.51 × 10^-7^	17.9	4.65
ER	17	9.45	-	-	N.S.	N.S.
PRC2	61	5.87	multicellar organismal development	4.90 × 10^-7^	N.S.	5.07
8q24	10	7.80	-	-	N.S.	N.S.
17q12	11	7.78	-	-	N.S.	N.S.

N.S. denotes 'not significant'.

Each expression module consists of dozens of genes. We performed Gene Ontology (GO) analysis to examine whether the obtained expression modules are enriched in genes involved in specific cellular activities (Table 1). The GO analysis showed that most of the expression modules deduced from *cis*-regulatory motifs and ChIP-chip data contain a significant number of genes sharing common GO terms, such as immune response and cell cycle. Thus, these transcriptional modules have the potential to function for specific cellular activities.

**Figure 2:**
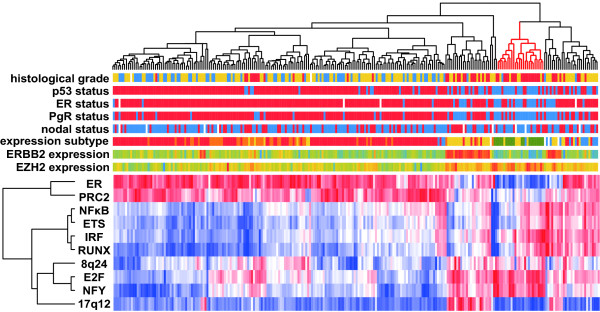
**Clustering analysis of expression module activity profiles in breast tumors**. Activity profiles of 10 expression modules extracted from breast cancer expression data were analyzed by hierarchical clustering. Red indicates increased activity and blue indicates decreased activity. The upper color bars indicate clinical and gene expression information of each tumor sample; histological grades (G1: red, G2: yellow G3: blue), p53 status (wildtype: red, mutant: blue), expression subtypes, ER, PgR and nodal status (positive: red, negative: blue), ERBB2 and EZH2 expression (increased expression: red, decreased expression: blue). The expression subtypes are based on the five major branches in the clustering dendrogram of the gene expression profiles. In the upper dendrogram, red branches represent a sample cluster which is enriched for triple negative breast tumors.

**Figure 3:**
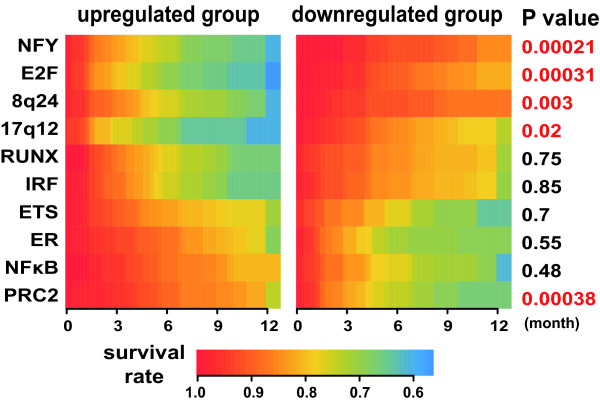
**Survival time analysis of expression module activity profiles in breast tumors**. Associations between survival time of patients and expression module activities were evaluated. Kaplan-Meier curves for two patient groups with different activities of each expression module were displayed using a color code (high survival rate: red, low survival rate: blue). P values are calculated for coefficients in Cox regression analysis.

The EEM analysis predicted the activity profiles of the 10 expression modules; we performed hierarchical clustering analysis of them as performed for ordinary gene expression profiles (Figure 2). We found that clustering of tumor samples based only on these 10 expression modules succeeded in dividing samples into several subtypes that are consistent with clinical information and gene expression profiles. This observation suggests that a significant degree of diversity of the breast cancer phenotypes can be explained by only these 10 expression modules. In other words, this result demonstrates that the EEM analysis successfully reduced gene expression data of extremely high dimension to the 10 components. We also performed survival time analysis and found expression modules associated with prognosis (Figure 3, see below).

The E2F and NFY expression modules show similar activity profiles, which are activated in high grade breast tumors and strongly correlated with poor prognosis. They also share common modules genes and appear to cooperatively regulate the cell cycle. Similar expression profiles shared by the RUNX ETS, IRF and NF*κ*B expression modules also suggest that these TFs regulate immune pathways cooperatively. These results are consistent with those of previous studies [32-36]. As expected, the ER expression module was found to be the most critical determinant of tumor subtypes. Their activity profiles are strongly correlated with ER status, demonstrating the validity of our approach. The 17q12 and 8q24 expression modules are derived from known amplified regions [15]. The 17q12 expression module contains the ERBB2 gene, while the 8q24 expression module contains genes residing near the Myc locus. The 17q12 expression is an important determinant of tumor subtypes and survival time. Although the 8q24 expression modules are not clearly associated with any subtypes, its upregulation is related to poor prognosis.

Triple-negative breast cancers characterized by a lack of the ER, progesterone receptor (PgR), and ERBB2 expression have attracted special attention in breast cancer research. In addition to their aggressive phenotype, they lack the benefit of specific therapy that targets these genes and, therefore, are associated with short survival. The sample cluster enriched for triple negative cancers has characteristic expression module activity profiles; the E2F and NFY expression modules are upregulated, while the ER, PRC2 and 17q12 expression modules are downregulated. Among them, the PRC2 expression module is especially intriguing. PRC2 is an epigenetic gene silencer, which plays a critical role in the maintenance of stem cells. They have also been reported to be implicated in neoplastic development. The PRC2 expression module was derived from gene sets bound by EED and SUZ12 and trimetylated at histone H3 lysin-27 in ES cells [12]. Therefore, our observation suggests similarity of transcriptional programs in both stem cells and the aggressive breast cancer. Triple-negative breast cancers are known to have poorly differentiated phenotype histology, which might be maintained by a PRC2-directed regulatory program. Recently a drug targeting PRC2 is developed [37]; PRC2 could be a therapeutic molecular target in triple negative breast cancers. Furthermore, we found that EZH2, a component of PRC2, belongs to the E2F expression module [38], while its expression is inversely correlated with profiles of the PRC2 expression module. This finding suggests that E2F-driven EZH2 overexpression is important for repression of the PRC2 expression modules in triple negative tumors. It should be noted that another independent study employing bioinformatics has also recently shown that PRC2 target genes are downregulated in malignant breast tumors, supporting our finding [39].

Inspection of individual genes in each expression module provided insights into regulatory networks in breast tumors. For example, our result suggests that auto-regulatory designs are prevailing in mammalian transcriptional networks. The E2F expression module contains three E2F family genes: E2F1, E2F7, and E2F8. The ER expression module also harbors ER itself and its interacting co-factor, FOXA1 [40]. Furthermore, RUNX3, one of the RUNX family genes, belongs to the RUNX expression module (see Additional File 1).

To obtain further insights into regulatory networks, we performed network analysis using protein-protein interaction (PPI) data in the HPRD [29]. Previous studies have demonstrated a significant correlation between yeast PPI and transcriptional networks [41,42]. This observation prompted us to examine whether human expression modules identified by EEM also tend to contain genes involved in the same protein complex. We calculated the Z score for the count of module gene pairs which interact directly (nearest neighbor pairs), and those which interact directly or via the next node of each (nearest or next-to-nearest neighbor pairs) (Table 1). This analysis showed that the transcriptional modules that were identified based on *cis*-regulatory motifs or ChIP-chip data harbor a statistically significant number of physically interacting pairs, revealing a tight coupling of transcriptional and PPI networks in mammalian cells.

**Figure 4:**
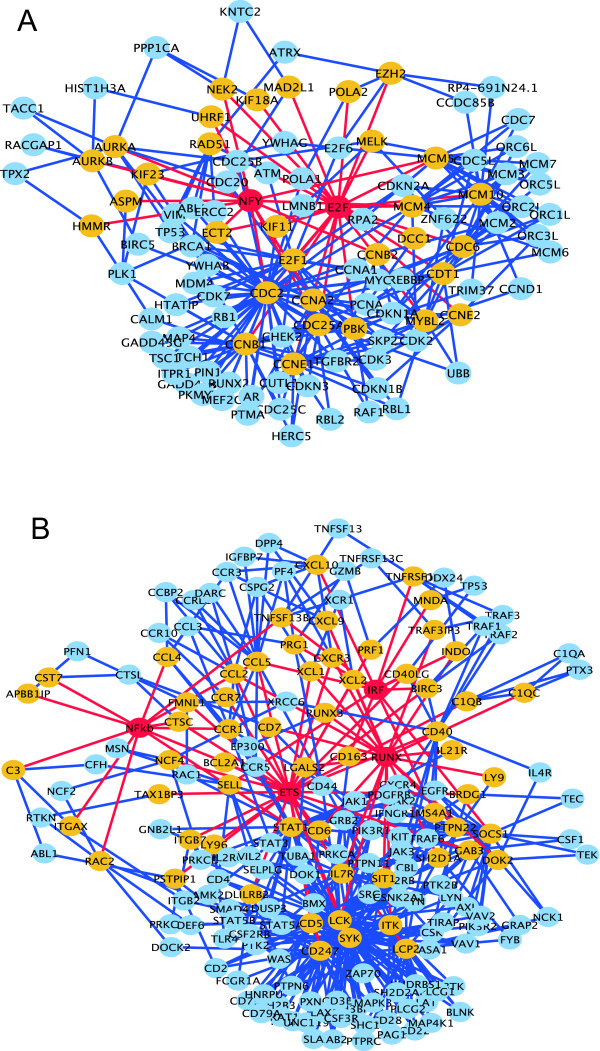
**PPI and transcriptional sub-networks in breast tumors**. (A) The E2F and NFY expression modules and an overlapping PPI sub-network. (B) The ETS, IRF, NF*κ*B, and Runx expression modules and an overlapping PPI sub-network. For clarity, we displayed nodes which have more than one links. Red, yellow and blue nodes denote transcriptional regulators, regulated genes, and their interacting partners, respectively. Red links denote transcriptional regulations predicted by our analysis, and blue links denote PPIs registered in the HPRD.

Finally, by extracting expression module genes and interacting partners from the PPI data, we depicted molecular circuits in breast tumors based on multiple lines of evidence: PPI, expression coherence, *cis*-regulatory motifs and ChIP-chip data (Figure 4). These network views revealed that E2F regulates cell cycle hub genes, such as CDC2 and Cyclins, in cooperation with NFY. The transcriptional sub-network involving RUNX, ETS, IRF, and NF*κ*B regulates immune circuits involving various chemokines and chemokine receptors, some of which have been reported to be involved in tumor growth, invasion, and metastasis [43]. For example, a recent study showed that CCL5 can induce metastasis of breast tumors [44]. Consistent with a previous report [45], EEM predicted that ETS, IRF and NF*κ*B transcriptionally control CCL5, suggesting that these TFs are responsible for CCL5-matiated metastasis. Human breast tumors are histologically complex and contain a variety of cell types in addition to the carcinoma cells. Hence, the transcriptional programs controlling these immune circuits could operate not in the carcinoma cells themselves, but in the tumor microenvironment. Indeed, CCL5 was reported to be secreted from mesenchymal stem cells. Also, many other module genes are known to be specifically expressed in immune cells such as lymphocytes and macrophages. On the other hand, we could also identify the IRF expression module in the transcriptome of breast cancer cell lines (see Additional File 1). Thus, the IRF expression module may function in the carcinoma cells. Recently, global expression profiling of distinct cell population in breast tumors has been attempted [46]. We expect that application of EEM to such data will clearly show cell type specific regulatory programs.

Previously, Segal et al. [47] also reported expression modules in cancer transcriptome. However, their method identifies functional modules based on relative up or down-regulation of module genes in each sample, which contrasts with our method that takes into account expression coherence across all samples. In this study, our method succeeded in reducing the expression profiles of thousands of genes to the activity profiles of 10 expression modules which explains a significant degree of diversity of the breast cancer phenotypes. It should be noted that the expression module activity profiles show some similarity with oncogenic pathway activity profiles depicted by Bild et al. [48]. Their Bayesian regression-based method learns signatures that can predict pathway activities of clinical tumor samples from microarray experiments using cell line models. By contrast, our method searches for coexpressed gene sets under common regulatory mechanisms using prescribed gene sets. Hence, these two methods are considered to be complementary to each other. In addition to clustering analysis, we applied survival analysis to the expression module activity profiles and succeeded in identifying expression modules associated with prognosis. While a number of studies have identified signature genes associated with prognosis in breast cancer [6], the result suggested that our approach is also useful to search for such signature genes. Based on EEM-deduced expression modules, we also predicted transcriptional regulatory networks in breast tumors. Although some previous studies have addressed reverse engineering problems of regulatory networks in cancer cells [49], they are only based on correlation or conditional independence of expression profiles. On the other hand, our method incorporates evidence of direct TF regulation. Collectively, we can say that EEM is a powerful module discovery method that provides various types of information essential for a deeper understanding of cancer transcriptomes.

As well as these notable advantages, our approach has several limitations. EEM assumes that module genes behave coherently across all samples. However, because gene regulatory programs are usually functional in specific contexts, it might be more appropriate to assume that module genes are assumed to behave coherently in only a subset of samples. Under the current assumption, we might fail to find tumor subtype-specific expression modules. It is also probable that different genes in a common module are controlled by different modes of a regulatory program (e.g., it is known that some TFs act as both activators and repressors, depending on target genes). Although current version of EEM cannot detect expression modules which show such complex patterns of expression profiles, future studies will improve the algorithm to overcome these limitation. Also, it should be noted that EEM uses the size of a coherent subset as an index of expression coherence. We use the arbitrary parameter *r *to specify the minimum degree of coexpression of coherent gene subsets. It is possible that EEM misses tightly coexpressed small modules or loosely coexpressed large modules, depending the values of *r*. In such a case, optimization of *r *based on Z scores will improve results. Recently, another gene set screening method based on a different index of expression coherence [50] was also reported. Comparison of different coherence indexes should be addressed in future studies.

To search for expression modules utilizing EEM, we prepared a collection of seed gene sets based on *cis*-regulatory motifs and ChIP-chip data. Therefore, comprehensiveness of our method depends on coverage of these data. Although a large number of motifs are already registered in databases, the quality and coverage seem to be incomplete. However, because several promising methods that are suitable for high-throughput determination of TF binding specificity have been devised [51,52], more accurate and comprehensive data of regulatory motifs are expected to be available soon. Furthermore, instead of ChIP-chip, a more high-throughput and cost-effective alternative, the ChIP-Seq technique has recently emerged [53,54]. It is expected that a great deal of TF binding site data will be produced by ChIP-Seq in the next decade. These increasing amounts of data will enable more global analysis in the near future. In this study, we assumed that each expression module is regulated by a single TF. However, combinatorial regulations by multiple TFs are known to be essential in mammalian regulatory networks. Combinatorial analysis will be enabled by constructing expression modules based on *cis*-regulatory information about multiple TFs. We will focus on this problem in future studies.

## Conclusion

We apply a new gene-set based module discovery method, EEM, to breast cancer microarray data, and revealed 10 principal expression modules in the breast cancer transcriptome. The subsequent analyses of expression module activity profiles and predicted regulatory networks demonstrated their importance in the pathophysiology of breast cancer. We believe that our method will be a powerful tool to decode gene regulatory programs in cancer transcriptomes.

## Authors' contributions

AN, TA, and HA designed research; AN performed research; AN, ADS and MQZ contributed new analytic tools; AN, TA, and SI wrote the paper; all authors read and approved the final manuscript.

## Appendix

A pseudocode for the optimization procedure in EEM is as follows:

1: **comment: ***E*_*M *_is the set of expression profiles for a seed gene set (*i.e*. some subset of available genes).

2: **comment: ***r *is the given radius value.

3: $C_{B_{1}}$ ⇐ ∅

4: **for all ***e*_*i *_∈ *E*_*M *_**do**

5:    $C_{e_{i}}$ ⇐ {*e*_*j *_∈ *E*_*M*_: *d*(*e*_*j*_, *e*_*i*_) <*r*}

6:    **if **|$C_{e_{i}}$| > |$C_{B_{1}}$| **then**

7:       *B*_1 _⇐ *e*_*i*_

8:       $C_{B_{1}}$ ⇐ $C_{e_{i}}$

9:    **end if**

10: **endfor**

11: *C*_*B *_⇐ ∅

12: *T *⇐ {*B*_1 _and the 9 profiles in $C_{B_{1}}$ closest to *B*_1_}

13: **for all triples **{*t*_1_, *t*_2_, *t*_3_} ⊂ *T ***do**

14:    *t *⇐ (*t*_1_, *t*_2_, *t*_3_)/3

15:    *C*_*t *_⇐ {*e*_*i *_∈ *E*_*M*_: *d*(*e*_*i*_, *t*) <*r*}

16:    **if **|*C*_*t*_| > |*C*_*B*_| **then**

17:       *B *⇐ *t*

18:       *G*_*B *_⇐ *C*_*t*_

19:    **end if**

20: **end for**

21: **return **(*B*, *C*_*B*_)

## Supplementary Material

Additional file 1 **Supplementary text**. supplementary discussions, tables and figures. Click here for file
