# Supplementary material for: Gene set-based module discovery in the breast cancer transcriptome
Source: BMC Bioinformatics. 2009 Feb 26;10:71. doi: 10.1186/1471-2105-10-71 (PMC2674431; doi:10.1186/1471-2105-10-71)
Supplement: Additional file 1 — Supplementary text. supplementary discussions, tables and figures. [file 1471-2105-10-71-S1.pdf]

Supplementary Discussion

EEM extracts a coherent subset from a seed gene set and evaluates functionality of the module based on the size of the coherent subset. This approach was motivated by the observation that a putative functional seed gene set often includes a large subset of genes which behave coherently. For example, a gene set having E2F binding motif(s) in their promoters, a ER-bound gene set suggested by a ChIP-chip experiment, and a gene set on the 17q12 locus all harbor a large coherent subset in breast cancer transcriptome (Supplementary Figure 1). EEM also assumes such a coherent subset as a true expression module. This strategy is verified by our GO analysis applied to both putative and EEM-extracted expression module. This analysis clearly shows that EEM-extracted expression modules scores smaller P values than seed gene sets (Supplementary Table 1). In other words, EEM-extracted expression modules harbor more functional genes than seed gene sets. Expression modules based on ChIP-chip data and locus information are omitted from this analysis. Since ChIP-chip and expression data are derived from different tissues, it is necessary to take into account tissue specificity to assess functional enrichment accurately. Expression modules based on locus information does not have functional enrichment, because these expression modules are derived from oncogenic mutational events, and do not have biological function.

We systematically applied EEM to the expression profile data published by Miller *et al.* [2]. Although we identified 10 expression modules in the breast cancer transcriptome, there are some point to be discussed.

Based on gene locus data, in addition two expression modules that are derived from the 17q12 and 8q24 locus, genes residing on 6p22 are coherently expressed. This locus includes the histone gene cluster; the observed expression coherence might result from coordinated transcriptional regulation. However, since the histone gene cluster includes highly homologous family genes, another possible reason is that mishybridization of microarray probes artificially leaded to the result. Taking into account the latter possibility, we omitted this locus in the subsequent analysis.

A previous ChIP-PET analysis [5] showed that a fraction of the genes which are bound by p53 have expression profiles correlating with p53 status of breast cancer. We also statistically tested whether the p53-bound genes function as expression modules. However, contrary to the previous report, our analysis could not identify any coherently expressed gene subset in the putative p53 expression module. Statistical evaluation of the expression modules is one of the notable features of EEM, which has not been performed in previous studies [1][5].

We identified the PRC2 expression module as a functional one in breast

tumors. However, it should be noted that, for calculation of the significant Z score, we used a larger radius parameter ($r = 0.10$) than those for the others. This means that the PRC2 expression module is less coherently expressed than the other expression modules. We speculate that this is because the mechanism of the PRC2 transcriptional program is different from those of other DNA binding TFs; PRC2 functions via histone modification and DNA methylation. Another possible reason is that the seed gene set is based on ChIP-chip data sampled from a different biological condition (i.e., ES cells).

For the obtained 10 expression modules, we evaluated the results of EEM using independent breast cancer microarray data [4]. Most of them scored significant Z scores (Supplementary Table 2). Although The Z score for the NFY expression modules was marginally significant, they include a significant number of cell cycle genes. The PRC2 expression module also did not score a significant Z value. However, clustering analysis showed that a significant fraction of PRC2-bound genes are downregulated specifically in the basal type cluster, which is enriched for triple negative breast cancer (Supplementary Figure 2). Based on this observation, we concluded that the NFY and PRC2 expression modules were confirmed for the independent data. We found that the detection power of EEM is dependent on the size of expression profile data. Since the size of this data is smaller than that of the data used in the text, the NFY and PRC2 expression module might fail to score significant Z scores. Reproducibility was also confirmed for clustering analysis of the expression module activity profiles (Supplementary Figure 3).

We identified the RUNX, ETS, IRF, and NFκB expression module, which are enriched for immune response genes. To test whether they function in carcinoma cells or surrounding immune cells, we performed EEM search using expression data of breast cancer cell lines [3]. We then identified IRF expression module in the transcriptome of breast cancer cell lines ($Z = 12.0$). Thus, the IRF expression module may function in the carcinoma cells. However, since the size of the data used is relatively small, it is possible that we could not detect expression modules that actually function in the cell lines.

References

[1] Bar-Joseph, Z. *et al.* (2003). Computational discovery of gene modules and regulatory networks, *Nat. Biotechnol.*, **21**, 1337-1342.

[2] Miller, L. D. *et al.* (2005). An expression signature for p53 status in human breast cancer predicts mutation status, transcriptional effects, and patient survival, *Proc. Natl. Acad. Sci. U. S. A.*, **102**, 13550-13555.

[3] Neve, R. M. *et al.* (2006). A collection of breast cancer cell lines for the study of functionally distinct cancer subtypes, *Cancer Cell*, **10**, 515-527.

[4] Vlieghe, D. *et al.* (2006). A new generation of JASPAR, the open-access repository for transcription factor binding site profiles, *Nucleic Acids Res.*, **34**, D95-D97.

[5] Wei, C. L. *et al.* (2006). A global map of p53 transcription-factor binding sites in the human genome, *Cell*, **124**, 207-219.

Supplementary Tables and Figures

Supplementary Table 1: GO analysis applied to putative and EEM-extracted expression modules

module ID	size of extracted modules	size of seed gene sets	the most enriched GO for extracted modules	P value for extracted modules	the most enriched GO for seed gene sets	P value for seed gene sets
ETS	47	240	immune response	4.55×10^{-15}	immune response	6.51×10^{-12}
IRF	37	240	immune response	6.19×10^{-12}	immune response	1.13×10^{-10}
E2F	34	240	cell cycle	1.98×10^{-20}	DNA metabolic process	6.91×10^{-11}
RUNX	30	240	immune response	2.57×10^{-11}	immune system process	3.08×10^{-9}
NFY	29	240	cell cycle	9.48×10^{-14}	M phase of mitotic cell cycle	4.35×10^{-9}

Supplementary Table 2: expression modules identified based on independent breast cancer microarray data

module ID	size	Z score	the most enriched GO	P value for the most enriched GO	Z score for nearest neighbor pairs	Z score for nearest or next to nearest pairs
ETS	48	10.9	immune system process	1.91×10^{-14}	15.1	7.50
IRF	34	6.72	immune system process	1.05×10^{10}	N.S.	N.S.
RUNX	33	6.41	immune system process	1.15×10^{-9}	12.5	7.94
E2F	26	4.26	cell cycle	3.34×10^{-15}	22.3	15.0
NFY	22	3.03	cell cycle	8.52×10^{-16}	21.2	14.0
NFkB	18	5.40	immune system process	4.12×10^{-5}	4.31	5.30
ER	15	4.95	-----	-----	N.S.	N.S.
PRC2	41	N.S.	-----	-----	-----	-----
8q24	22	16.5	-----	-----	N.S.	N.S.
17q12	9	8.45	-----	-----	N.S.	N.S.

N.S. denotes 'not significant' .

Supplementary Table 3: genes that compose the 10 expression modules in the breast cancer transcriptome

17q12

THRAP4
GSDML
FBXL20
PSMD3
STARD3
ERBB2
GRB7
C17orf37
PNMT
PERLD1
CRKRS

8q24

PYCRL
C8orf55
SLC39A4
RECQL4
MFSD3
BOP1
EXOSC4
DGAT1
LOC727765
GPR172A

E2F

POLA2
LOC728688
UHRF1
RAD51
MCM10
CDC6
MCM5
GINS1
MYBL2
E2F1
MCM4
CCNE1
NUDT1
EZH2
ECT2
NEK2
KIF18A
E2F8
CCNB1
DCC1
GGH
NUSAP1
KIF11
DTL
CDC25A
MAD2L1
MELK
E2F7
CDT1
PBK
CDC2
EXO1
CCNE2
GPR19

ER

SEMA3B
CA12
KIAA1467
ESR1
HPN
AGR2
STC2
MLPH
SLC19A2
JMJD2B
FOXA1
ANXA9
TFF1
LOC124220
FBP1
BCMP11
GREB1

ETS

BIRC3
BRDG1
LCP2
P2RX5
TAX1BP3
LAG3
LYZ
NCF4
CD40
CD40LG
GIMAP1
GIMAP2
CCL2
CTSC
CRTAM
CD5
BIN2
LRMP
ARHGAP9
CCR7
LILRB2
LOC652626
EPSTI1
GIMAP4
PARVG
ITGB7
PSTPIP1
BCL2A1
C1orf162
XCL1
XCL2
LY96
GPR114
LOC654091
GAB3
CCL5
ARHGAP25
SYK
TMC8
IL7R
C1QB
PRF1
SLC9A9
LCK
FMNL1
CXCR3
CD247

IRF

BIRC3
TNFRSF17
CD40LG
TNFSF13B
GIMAP1
GIMAP2
SLC15A3
BTN3A3
IFIH1
GBP1
LRMP
IFIT3
PRG1
ZBP1
INDO
EPSTI1
RTP4
IFI44
GZMA
CD96
CLIC2
MS4A1
LOC648998
NCF1
SLAMF8
CCL5
GBP4
ARHGAP25
MNDA
LOC648998
CXCL10
RNASE6
CD163
SOCS1
C5orf20
BTN3A2
SELL

NFY

LOC728688
UHRF1
ASPM
HMMR
GTSE1
UBE2T
AURKA
MCM5
MCM4
UBE2S
TTK
KIF20A
ECT2
NEK2
KIF23
C15orf42
CCNA2
FAM54A
CCNB2
TMSL8
RECQL4
CDC25A
CDC2
AURKB
CDCA2
LOC652645
LOC653820

NFkb

APBB1IP
CST7
ARHGAP4
TNFSF13B
CD37
NKG7
CTSC
CD69
STAT1
C3
LOC653879
RAC2
CCL4
BCL2A1
ITGAX
SLAMF8
CCL5
AIM2
CCR1

PRC2

ABCC8
PTGER3
CBLN4
SIDT1
SPAG6
LRP2
PGR
CHRD
COMP
GATA3
SORCS1
HOXB6
PMP22
ZBTB16
SLC1A2
DLX2
KIAA1324
IGSF21
HOXB3
GRIA2
DUSP4
TBX2
C20orf103
CITED1
ADCY4
KLF4
SEMA6D
SLC27A2
CXCL14
ASTN2
ESAM
ADRA2A
STK32B
PGM5
DKK2
KCNMA1
CACNA1D
CLSTN2
BTG2
CAMK2N1
UCN
FBP1
DACH1
NPNT
REPS2
PRKCE
HOXB2
PODN
SCN4B
JUN
DPY19L2
ERBB4
GATA2
EGR3
ZADH2
C3orf15
MAPT
ESPN
COL4A5
PTPRT
FLJ45983

RUNX

TRAF3IP3
CD6
RUNX3
LYZ
LGALS2
IL21R
CCL2
CD69
BIN2
ITK
PSCDBP
GNLY
LY9
INDO
ADAMDEC1
PTPN22
AOAH
SIT1
CXCL9
XCL1
XCL2
GZMA
DOK2
C1QC
IL7R
CD7
SLC9A9
LCK
SH2D1A
CXCR3

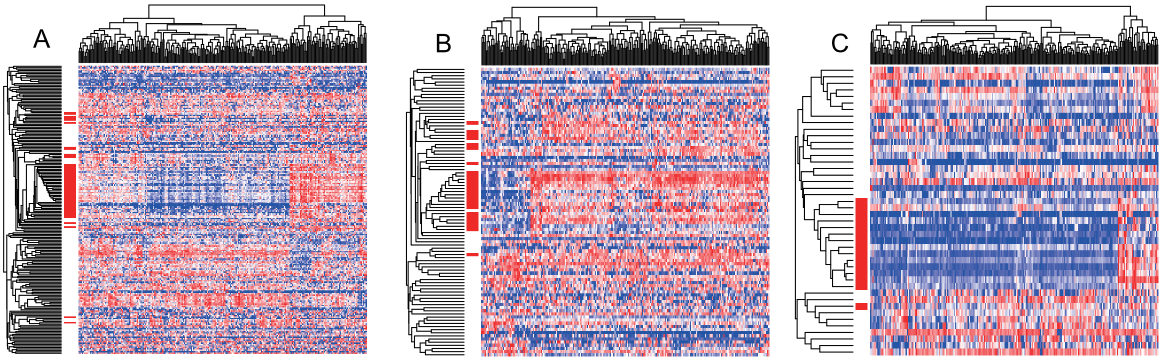

Supplementary Figure 1: Clustering analysis of putative functional seed gene sets using breast cancer microarray data. We performed hierarchical clustering analysis of expression profiles for genes with E2F binding motif(s) in their promoters (A), ER-bound genes suggested by a ChIP-chip experiment (B), and genes on the 17q12 locus (C). Expression profile matrices with their rows for genes and their columns for samples are displayed using a color code (red: high expression, blue low expression). Red bands between the expression profile matrices and the gene dendrograms indicate coherent gene subsets extracted by the EEM algorithm.

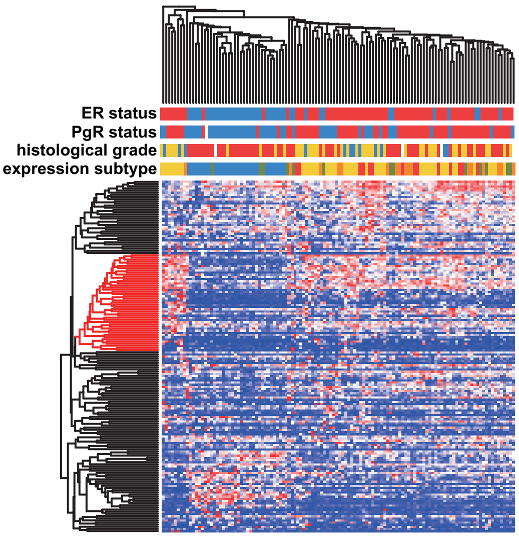

Supplementary Figure 2: Clustering analysis of PRC2-bouned genes using independent breast cancer microarray data. For genes bound by PRC2 in ES cells, expression profiles in breast tumors were analyzed by hierarchical clustering. Red indicates increased activity and blue indicates decreased activity. Upper color bars indicate sample information accompanying the microarray data; ER and PgR status (positive: red, negative: blue), histological grade (G1: red, G2: yellow G3: blue), and the expression subtypes based on the gene expression profiles (basal: blue, ERBB2: green, luminal A: yellow, luminal B: orange, normal: red). In the gene dendrogram, red branches represent a gene cluster repressed specifically in basal subtype tumors.

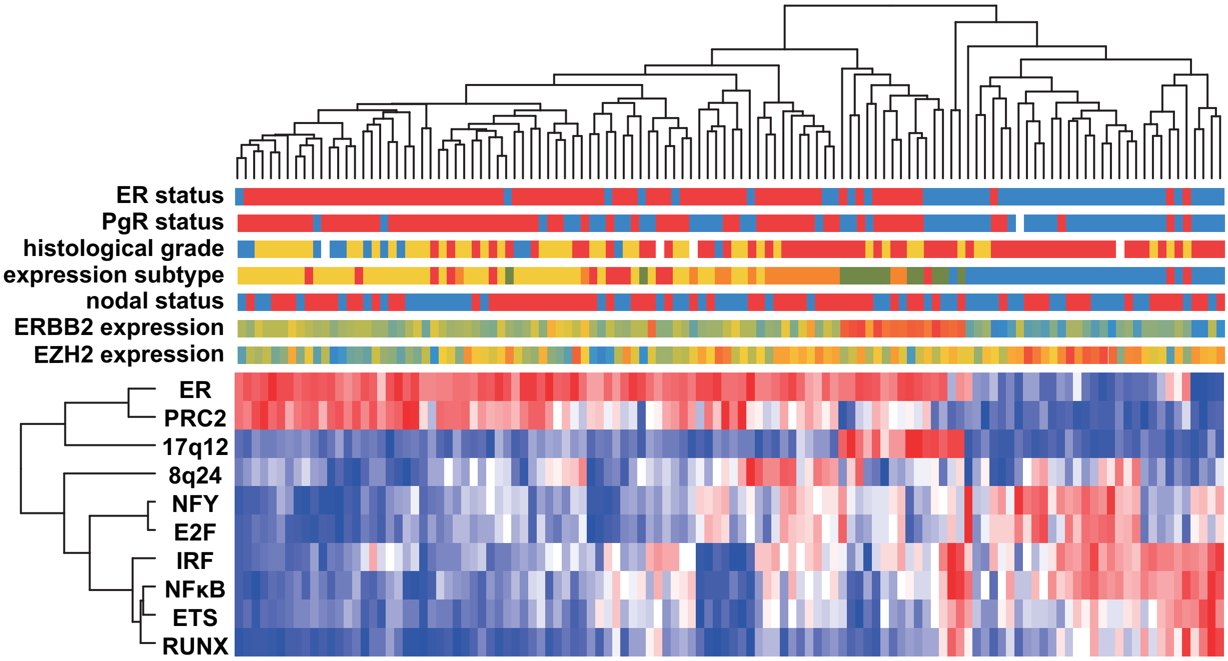

Supplementary Figure 3: Clustering analysis of expression module activity profiles using independent breast cancer microarray data. Activity profiles of 10 expression modules extracted from breast cancer expression data were analyzed by hierarchical clustering. Red indicates increased activity and blue indicates decreased activity. Upper color bars indicate clinical information and gene expression profiles; histological grade (G1: red, G2: yellow G3: blue), the expression subtypes based on the gene expression profiles (basal: blue, ERBB2: green, luminal A: yellow, luminal B: orange, normal: red), ER, PgR and nodal status (positive: red, negative: blue), ERBB2 and EZH2 expression (increased expression: red, decreased expression: blue).
